# Anti-folate drug resistance in Africa: meta-analysis of reported dihydrofolate reductase (*dhfr*) and dihydropteroate synthase (*dhps*) mutant genotype frequencies in African *Plasmodium falciparum *parasite populations

**DOI:** 10.1186/1475-2875-9-247

**Published:** 2010-08-30

**Authors:** Sankar Sridaran, Shannon K McClintock, Luke M Syphard, Karen M Herman, John W Barnwell, Venkatachalam Udhayakumar

**Affiliations:** 1 Malaria Branch, Division of Parasitic Diseases and Malaria, Center for Global Health, Centers for Disease Control and Prevention, 1600 Clifton road NE, Mail Stop D-67 Atlanta, GA, 30333, USA; 2 Atlanta Research and Education Foundation, VAMC, Atlanta, GA 30033, USA

## Abstract

**Background:**

Mutations in the dihydrofolate reductase (*dhfr*) and dihydropteroate synthase (*dhps*) genes of *Plasmodium falciparum *are associated with resistance to anti-folate drugs, most notably sulphadoxine-pyrimethamine (SP). Molecular studies document the prevalence of these mutations in parasite populations across the African continent. However, there is no systematic review examining the collective epidemiological significance of these studies. This meta-analysis attempts to: 1) summarize genotype frequency data that are critical for molecular surveillance of anti-folate resistance and 2) identify the specific challenges facing the development of future molecular databases.

**Methods:**

This review consists of 220 studies published prior to 2009 that report the frequency of select *dhfr *and *dhps *mutations in 31 African countries. Maps were created to summarize the location and prevalence of the highly resistant *dhfr *triple mutant (N51I, C59R, S108N) genotype and *dhps *double mutant (A437G and K540E) genotype in Africa. A hierarchical mixed effects logistic regression was used to examine the influence of various factors on reported mutant genotype frequency. These factors include: year and location of study, age and clinical status of sampled population, and reporting conventions for mixed genotype data.

**Results:**

A database consisting of *dhfr *and *dhps *mutant genotype frequencies from all African studies that met selection criteria was created for this analysis. The map illustrates particularly high prevalence of both the *dhfr *triple and *dhps *double mutant genotypes along the Kenya-Tanzania border and Malawi. The regression model shows a statistically significant increase in the prevalence of both the *dhfr *triple and *dhps *double mutant genotypes in Africa.

**Conclusion:**

Increasing prevalence of the *dhfr *triple mutant and *dhps *double mutant genotypes in Africa are consistent with the loss of efficacy of SP for treatment of clinical malaria in most parts of this continent. Continued assessment of the effectiveness of SP for the treatment of clinical malaria and intermittent preventive treatment in pregnancy is needed. The creation of a centralized resistance data network, such as the one proposed by the WorldWide Antimalarial Resistance Network (WWARN), will become a valuable resource for planning timely actions to combat drug resistant malaria.

## Background

### The malaria problem

Global malaria control initiatives have a turbulent history driven by a number of biological, social, and economic factors. The Malaria Eradication Programme launched by the World Health Organization (WHO) in 1955 successfully eliminated malaria in many temperate and sub-tropical regions of the world, but little progress was made in sub-Saharan Africa. The programme confronted technical challenges including, but not limited to: anti-malarial drug resistance, insecticide resistance, lack of infrastructure to implement and sustain control measures, and a lack of community engagement and participation [1]. These obstacles collectively damaged the long-term sustainability of the eradication programme, which was ultimately abandoned in 1969 [2].

The renewed vision for malaria control and eventual elimination focuses on the development of new tools and improved integration of existing interventions [3]. Financing from the Global Fund to Fight AIDS, Tuberculosis, and Malaria; the World Bank Malaria Booster Program; and the U.S. President's Malaria Initiative (PMI) have increased funding for malaria control. One objective of the PMI includes a 50% reduction in malaria mortality in 15 African countries over a five-year period by expanding the coverage of effective interventions including: insecticide-treated mosquito nets (ITNs), indoor residual spraying with insecticides, intermittent preventive treatment for pregnant women (IPTp), and artemisinin-based combination therapy (ACT) [3,4].

ACT is a vital component of the malaria control strategy and currently represents the last line of defense against infections that have developed resistance to previous anti-malarial therapies. Chloroquine (CQ) monotherapy was highly effective in treating malaria for several years, but eventual appearance of CQ resistance to *P. falciparum *became a major challenge for control programs [5-8]. The onset of pervasive CQ resistance led to its replacement with anti-folate drugs, most notably sulphadoxine-pyrimethamine (SP), as a first-line therapy in many parts of the world. However, resistance to anti-folate drugs emerged rapidly. ACT has since been introduced to either work in combination with anti-folates or replace them as first-line therapies in many settings, reviewed in [9,10].

### Monitoring anti-malarial drug resistance

Drug resistance continues to present a major threat to remaining effective anti-malarial therapies, such as ACT, that currently serve as a cornerstone of the malaria control strategy. Therefore, the success of control efforts relies heavily upon surveillance methods to adequately monitor and promptly respond to emerging resistance.

Clinical efficacy trials that measure the therapeutic failure rate following administration of a particular drug are the gold-standard method for gauging the effectiveness of anti-malarial therapies. However, clinical efficacy trials are resource-intensive, making them difficult to use broadly for surveillance purposes, reviewed in [11]. Translating a measure of drug efficacy into a reliable assessment of drug resistance is also obscured by a number of host, drug, and parasite factors such as: acquired immunity [12], compliance dosing [13], nutritional status [14], pharmacokinetics [15], parasite synchronicity and biomass [16], and endogenous parasite resistance mechanisms, reviewed in [17]. Therefore, studies that focus on specific interactions among host, drug, and parasite can make costly clinical trials more informative by helping to better differentiate and identify the role of drug resistance.

Pharmacological and *in vitro*-based assays have been used as tools to examine the role of host-drug and parasite-drug interactions that influence clinical efficacy of anti-malarials. Specifically, pharmacological studies conducted within the context of clinical trials can enable an examination of pharmacokinetics and pharmacodynamics (PK-PD) and their role in therapeutic response. An understanding of PK-PD in different clinical settings and populations is essential to establish therapeutic drug dosages for target groups and distinguish between true therapeutic failures and other factors such as poor adherence, drug quality, absorbance, and elimination. PK-PD data is also essential if outcomes from different clinical efficacy trials are to be accurately compared.

*In vitro *parasite susceptibility assays enable a measure of intrinsic chemosensitivity of *P. falciparum *parasites to a particular drug by controlling for host factors such as acquired immunity, nutritional status, and compliance dosing. Most *in vitro *studies report the 50% inhibition concentration (IC_50_) of an anti-malarial drug, which in turn serves as an indicator of parasite tolerance to the drug. *In vitro *assays offer benefits, such as the ability to quantify the parasite inhibiting properties of partner drugs within combination therapies and identify temporal trends by comparing the drug sensitivity profiles of frozen reference parasite lines with samples in question. Both *in vitro *and pharmacological studies play an important role in differentiating drug resistance from other potential causes of decreased drug efficacy. Although these methods complement clinical efficacy trials to collectively identify the presence of drug resistance and help quantify its impact on drug efficacy, they do little to propose specific parasite resistance mechanisms.

Molecular studies examining genetic correlates of resistance differ in scope from *in vitro *and pharmacological studies in that they provide the means to identify genetic markers that confer resistance. Association and genetic complementation studies have identified and validated genetic markers of resistance for older anti-malarial drugs, such as CQ, SP and chlorproguanil-dapsone [18-24]. Molecular surveillance studies that examine the prevalence and spread of these validated markers present a practical means of monitoring dynamic drug resistant parasite populations in different parts of the world and could be useful in planning drug efficacy studies [25]. Lessons learned from the identification and epidemiological use of molecular markers of resistance to formerly efficacious anti-malarials will be useful for the improvement of molecular surveillance capabilities for anti-malarials currently in use, such as ACT.

Clinical, pharmacological, *in vitro*, and molecular studies each shed light on a certain aspect of drug efficacy and drug resistance, but each also has its own set of benefits and limitations. Lack of standardization across studies of each type and differences in conventions for reporting data are two pervasive problems that seriously limit the ability to compare studies conducted at different times and locations. In an effort to mitigate these and other issues, the WorldWide Anti-malarial Resistance Network (WWARN) has put forth a comprehensive plan to network clinical, *in vitro*, pharmacological, and molecular studies in a centralized database. The proposed structure and objectives for each of the four components of the database have been detailed in a series of articles [11,26-28].

The need to standardize and network molecular data is particularly pressing. The advancement of high-throughput molecular techniques has enabled a rapid increase in the number of molecular studies conducted in recent years; however, a lack of meaningful ways to compare studies severely limits the potential utility of this valuable information. Databases that assemble the reported frequency of resistant parasite genotypes from multiple molecular studies can provide more comprehensive and easily accessible information. Drug resistance maps at the London School of Tropical Medicine and Hygiene [29] has assembled the reported frequency of mutations associated with SP resistance across Africa. The site includes data from approximately 100 studies in an open-access map-based format with electronic links leading to the articles from which the data was derived. More recently, Picot *et al *[30] used a meta-analysis to show significant association between the presence of established genetic markers of resistance and increased therapeutic failure rates for corresponding anti-malarial drugs across a total of 92 studies conducted worldwide. Unfortunately, very few studies of the total number available in the literature met criteria for data linkage in this meta-analysis and had to be excluded from the study.

A growth in the number of molecular studies reporting the frequency of known resistance markers presents new opportunities to assess the broader impact that reporting conventions and study methodology may be having on the data being produced. Some of the variables that have been proposed as potentially influential include age and clinical status of the sampled population, date and location of the molecular study, reviewed in [31], and reporting conventions for polyclonal infections [32]. No previously published meta-analysis has ever used congregated molecular data to quantitatively assess the impact these variables may be having on the reported frequency of mutations that confer anti-malarial drug resistance. Therefore, an empirical database that not only summarizes molecular studies but also assesses the impact of potentially influential variables can provide a basis for standardization and help advance objectives proposed by WWARN for the molecular component of a centralized surveillance database.

### Anti-folate resistance database

Resistance to anti-malarial drugs for which validated molecular markers are available is already widespread; however, a database that specifically assembles studies reporting the frequency of parasite genotypes associated with anti-folate resistance is valuable for a number of reasons.

First, SP use in combination with artemisinin-based derivatives [33,34] and as a monotherapy for intermittent preventive therapy (IPT) in pregnant women [35] continues in parts of Africa and has demonstrated clinical efficacy, even in areas where SP is no longer used to treat acute malaria in the general population due to resistance. Clinical trials in these areas also suggest SP IPT can have a protective effect against the risk of clinical malaria and malaria anemia in infants [36], but in some cases this has been associated with higher risk of severe malaria rebound [37]. The prevalence and role of SP resistant parasite genotypes in these populations must be evaluated since this information may be useful in complementing clinical studies that inform drug policy changes. Second, there are multiple anti-folate drugs currently being administered in Africa with the same drug targets. Trimethoprim-sulphamethoxazole, administered as Cotrimoxazole^® ^(CTX), is a low cost regimen that has been shown to decrease morbidity and mortality in HIV-infected individuals [38]. However, CTX shares the same drug targets as SP to decrease the incidence of illness caused by bacterial and protozoan infections [39]. Thus, there is a concern that widespread administration of CTX may promote cross-resistance to SP. Although a recent study did not find any rapid rise in the antifolate-resistant *P. falciparum *genotypes following CTX treatment [40], continued surveillance is necessary to assess the long term trends. Chlorproguanil-dapsone is increasingly being used in Africa for treating malaria. It has the same drug targets as SP, but has shown to be more active in killing parasites *in vitro *[41] and has been more effective in treating malaria than SP *in vivo *[42,43], even when SP resistant parasite genotypes are prevalent in the population [44,45]. Monitoring anti-folate resistant parasite genotypes in populations receiving CTX prophylaxis or chlorproguanil-dapsone is critical to better understand the role these drugs may have in inducing or curbing cross-resistance.

### Anti-folate drug action and resistance mechanisms

The anti-folate class of drugs consists of compounds that bind enzymes necessary for parasite folate biosynthesis. The most widely used anti-malarial drugs within this class are sulphadoxine-pyrimethamine (SP) and, more recently, chlorproguanil-dapsone. The pyrimethamine portion of SP and chlorcycloguanil, the active metabolite of chlorproguanil, bind the enzyme dihydrofolate reductase (DHFR) [46]. Sulphadoxine and dapsone bind the enzyme dihydropteroate synthase (DHPS) [45,47,48]. Both enzymes are part of the parasite folate synthetic pathway and the inhibition of these enzymes leads to decreased production of tetrahydrofolate [49]. Tetrahydrofolate is a necessary cofactor for the production of a number of folate precursors including dTMP and methionine. Decreased production of these precursors severely inhibits the *Plasmodium *parasite life cycle [50].

Mutations in the *dhfr *and *dhps *genes of *P. falciparum *parasites have been associated with decreased parasite sensitivity to the anti-folate drugs. A change from wildtype Ser108 to Asn108 (S108N) in *dhfr *is sufficient to cause low level pyrimethamine resistance both *in vitro *and *in vivo *[18,21,51]. This single mutation can increase *in vitro *resistance to pyrimethamine by 100-fold relative to wild-type [21]. The progressive addition of mutations altering Cys50 to Arg (C50R), Asn51 to Ile (N51I), Cys59 to Arg (C59R), and Ile164 to Leu (I164L) in *dhfr *can yield higher levels of SP resistance *in vitro *and *in vivo *[51-56]. Genotypes consisting of multiple mutations in the *dhfr *gene have evolved in different parts of the world (see Table 1) and are most often associated with higher levels of resistance than the single mutant genotypes. The triple *dhfr *mutant genotype consisting of N51I, C59R, and S108N shows *in vitro *resistance to pyrimethamine that is 225 times higher than a wildtype lab strain [41,52] and has demonstrated strong association with *in vivo *SP treatment failure [32,57,58]. The Ala16 to Val (A16V) and Ser108 to Thr (S108T) mutations in *dhfr *confer resistance to cycloguanil but not pyrimethamine [19,20]. The addition of the N51I, C59R, and I164L mutations in the presence of S108N confers high levels of resistance to both pyrimethamine and cycloguanil [19,20,56].

**Table 1:** Mutant genotypes associated with high levels of anti-folate resistance

*dhfr *genotypes	Location	Reference
N51I/C59R/S108N	Asia, Africa	Reviewed in [17]
N51I/C59R/S108N/I164L	Asia, Africa	Reviewed in [17]; [88]
N51I/S108N/I164L	South America, Africa	[96]; [97]; [98]; [88]
C50R/N51I/S108N	South America	[96]
		
***dhps *genotypes**	**Location**	**Reference**

A437G/K540E	Africa, Asia, Indonesia	Reviewed in [17]; [99]; [100]
S436A/A437G	Africa	[101]; [79]
A437G/A581G	South America	[102]
A437G/K540E/A581G	Asia, South America	[96]; [103]; [97]
S436A/A437G/K540E/A581G; S436A/A437G/K540E	Asia	[104]; [105]

Mutations in codons Ser436 to Ala or Phe (S436A/F), Ala437 to Gly (A437G), Lys540 to Glu (K540E), Ala581 to Gly (A581G), and Ala613 to Ser or Thr (A613S/T) in *dhps *have been shown to affect parasite susceptibility to the sulpha drugs including sulphadoxine and dapsone [45,59,60]. The *dhps *A437G mutation alone predicted clinical failure of SP in parts of Kenya [61]. Data from various malaria endemic areas suggest asymmetric selection of resistant genotypes starting with mutations in *dhfr *and followed by those in *dhps *[50]. Multiple mutant *dhps *genotypes have also evolved in different parts of the world (see Table 1). The double *dhps *mutant genotype consisting of A437G and K540E is highly associated with *in vivo *clinical failure independently [58,62]. However, the quintuple mutant genotype consisting of the double *dhps *mutant genotype (A437G, K540E) in combination with the *dhfr *triple mutant genotype (S108N, N51I, C59R) is a better predictor of clinical failure than either multiple mutant genotype alone [58,63].

### Objectives

The objective of this meta-analysis are to 1) provide a comprehensive database of reported frequencies for resistant *dhfr *and *dhps *mutant genotypes in African *P. falciparum *populations; 2) highlight the significance and limitations of these studies; 3) use condensed tables, plots, and geostatistical illustrations to present frequency data; 4) use statistical models to describe changes in the prevalence of select highly resistant genotypes over the years; and 5) elucidate the impact of variables that may influence reported genotype frequencies.

## Methods

### Literature search

A literature search was done using the National Library of Medicine Pubmed/Medline search engine. Search terms included "dihydropteroate synthase," "*dhps*," "dihydrofolate reductase," "*dhfr*," "*Plasmodium falciparum*," "Africa," "sulphadoxine pyrimethamine," "antifolate," "drug resistance," "Fansidar." Boolean operators were used to search for articles using these terms in various combinations. All variations in the spelling of these terms were accounted for in the search. Additional articles were found through the Related Articles search feature on the Pubmed website and through the reference sections of candidate articles. The Drug Resistance Maps database [29] was also used to identify studies that may have been missed in the Pubmed/Medline search. Each article was then individually screened to identify those that met the following criteria:

1) The article must have been published in English prior to Jan 1^st^, 2009 to be included.

2) The article must explicitly state or provide interpretable mutation frequencies for any of the selected codons in *dhfr *(16, 50, 51, 59, 108) and/or *dhps *(436, 437, 540, 581, 613) to be included.

3) The article must have provided mutant frequency data derived from pre-treatment blood isolates to be included. Genotype frequency data derived from patients receiving anti-malarial drugs within two weeks of enrollment and/or patients who were determined to have therapeutic levels of anti-folate drugs in their bloodstream at the time of enrollment were excluded. Genotype frequency data derived from *in vitro *cultured blood isolates were also excluded.

4) The study must have restricted its sampling to blood isolates of African origin from local patient populations to be included. Genotype frequency data derived from travelers were excluded.

### Mutant allele frequency (MAF) and mutant genotype frequency (MGF) databases

Data reporting the frequency of mutations in *dhfr *and *dhps *were extracted from published articles and were summarized using two separate databases called the Mutant Allele Frequency (MAF) database and Mutant Genotype Frequency (MGF) database (see Additional Files 1 &2, respectively). The MAF database reports the frequency of mutations in *dhfr *and *dhps *for each codon individually. Whereas, the MGF database describes the frequency of the highly resistant *dhfr *triple mutant genotype (N51I, C59R, S108N) and *dhps *double mutant genotype (A437G, K540E) using a standardized system, similar to the format described by Kublin *et al *[32]. Table 2 has been condensed to describe how genotypes were categorized in the MGF database.

**Table 2:** *dhfr *and *dhps *genotype classification

	*dhfr *codon				*dhps *codon	
	**N51I**	**C59R**	**S108N**		**A437G**	**K540E**

						

Single	X			Single	X	

		X				X

			X			

						

Double	X	X		Double	X	X

		X	X			

	X		X			

						

						

Triple	X	X	X			

Each row represents a mutant genotype for either the *dhfr *gene or *dhps *gene. A white box represents a wildtype codon whereas a box with an "X" represents a mutant codon. The MGF database (Additional File 2) reports the frequency of mutant genotypes using the single, double, and triple mutant categories for *dhfr *and the single and double mutant categories for *dhps*.

One of the major methodological differences across studies was the reporting standards for mixed mutant genotypes. Mixed mutant genotypes are detected when two or more populations of parasites with distinct genotypes at particular codons of interest are present in a single infection. These infections are commonly referred to as "polyclonal" since multiple clonal populations of parasites are present in a single blood isolate. Varying classifications for mixed genotypes can have a significant impact on the determination of mutant genotype frequency; particularly in high transmission settings where polyclonal infections have been reported in greater than 50% of sampled isolates, reviewed in [64].

A majority of studies included in this review used the Restriction Fragment Length Polymorphism (RFLP) technique to differentiate between the mutant and wildtype form at codons of interest. Since this is an indirect method to detect codon change it has some inherent limitations in identifying appearance of new mutations and in accurately genotyping mixed infections [65]. A number of more recent studies use different methods to detect mutant genotypes including pyrosequencing, direct sequencing, and RT-PCR methods. However, each of these methods can have a different sensitivity and specificity in detecting various mutations accurately and thus can contribute to some variation in estimating the frequency of mutant genotypes. Since there is not a universal convention for determining and reporting different mutants, it was a challenge to compare different studies. Therefore, a rubric (Table 3) was created to categorize various reporting standards and establish a standardized approach to extract mutant genotype frequency data. The categories within the rubric are: combined, excluded, separate, split, and N/A. The combined category was used to describe studies that reported mixed mutant genotypes as pure mutants. The excluded category was used to describe studies that completely excluded mixed genotypes from the analysis. The separate category was used to describe studies that reported the mixed genotype frequency separate from the pure mutant or pure wildtype genotype frequencies. For this review, mixed genotype frequencies were reported separately from the pure mutant or pure wildtype genotype frequencies in the MGF database whenever possible. The split category was used to describe studies that considered mixed genotypes to be both mutant and wildtype; therefore, they were split between both. For this review, mixed genotypes were only split in the MAF database whenever possible. Finally, the N/A category was used to describe studies that made no mention of mixed genotypes when reporting genotype frequency. The mixed mutant category, year of study, age description of sampled population, and frequency data corresponding to each study can be found in the MAF and MGF databases. The ID number for each study links directly to a citation for the article from which the data was extracted (see Additional File 3). Calculations used to determine the mutant, wildtype, and mixed frequencies for studies within each category are shown in Table 3.

**Table 3:** Genotype frequency calculation rubric

	Mutant Frequency =	Wildtype Frequency =	Mixed Frequency =
**Combined**	(Mu + Mi)/(Mu + Mi + Wt)	Wt/(Mu + Mi + Wt)	N/A
**Excluded**	Mu/(Mu + Wt)	Wt/(Mu + Wt)	N/A
**Separate**	Mu/(Mu + Wt + Mi)	Wt/(Mu + Wt + Mi)	Mi/(Mu + Wt + Mi)
**Split (MAF only)**	(Mu + Mi)/[Mu + Wt + (2 × Mi)]	(Wt + Mi)/[Mu + Wt + (2 × Mi)]	N/A
**N/A**	Mi not described. Mutant frequency reported directly from study	Mi not described. Wildtype frequency reported directly from study	N/A

Mu = number of isolates carrying pure mutant genotype

Mi = number of isolates carrying mixed mutant genotype

Wt = number of isolates carrying pure wildtype genotype

### Maps and Time plots of *dhfr *triple mutant and *dhps *double mutant genotypes

**Figure 1:**
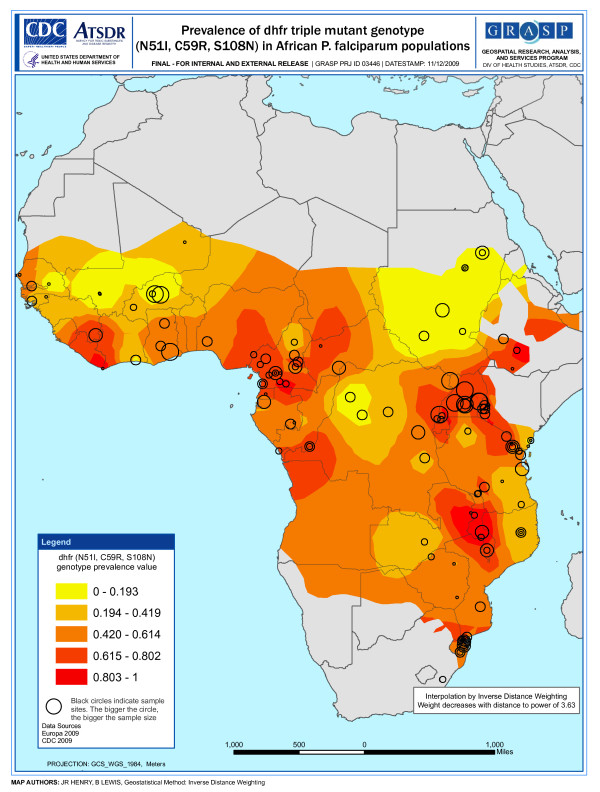
**Snapshot of prevalence - *dhfr *triple mutant genotype (N51I, C59R, S108N)**.

**Figure 2:**
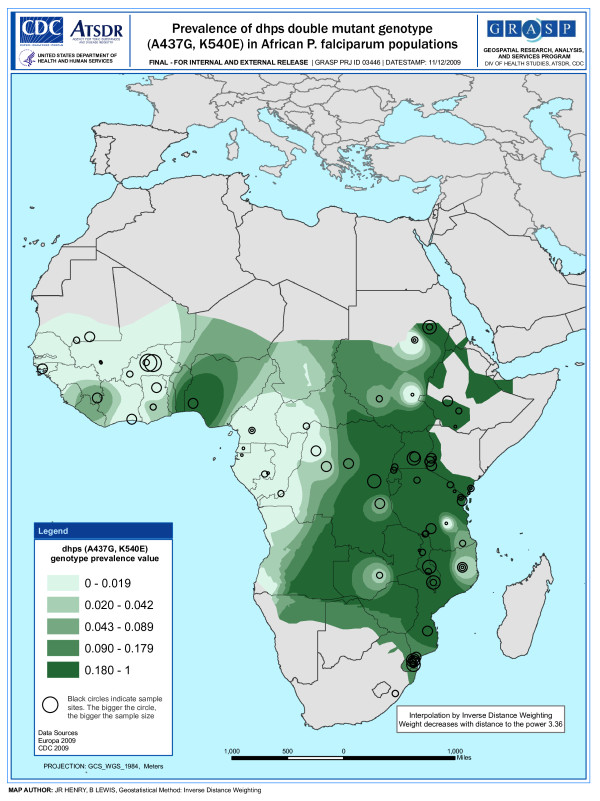
**Snapshot of prevalence - *dhps *double mutant genotype (A437G, K540E)**.

Maps describing the distribution and prevalence of the *dhfr *triple mutant genotype and the *dhps *double mutant genotype (see Figures 1 and 2, respectively) were created using ArcGIS 9.3.1 software. Locality coordinates were determined by searching for locality names in the Europa 2009 coordinate database. This database contains approximately 300,000 points with coordinate data along with identifiable geographical features and political boundaries. A majority, 75%, of the study sites were found in Europa. The remaining 25% could not be found; therefore, a search of academic journals through the Pubmed/Medline databases was conducted to find articles that describe these localities along with maps or coordinate information. The academic journal search yielded accurate locality descriptions for a majority of the remaining 25%; however, a few studies (~5% of the total) were only described by their proximity to political boundaries or geographical features such as rivers, mountains, or valleys. The extent error associated with these localities is greater, but was considered sufficient for the scale of the continent-wide map. These methods yielded geographic coordinates for each study. Coordinates were plotted using a black circle denoting the location of the study. The size of the circle is proportional to the number of samples examined.

An inverse distance weighted (IDW) algorithm was used to provide a snapshot of *dhfr *triple and *dhps *double mutant genotype prevalence in parts of Africa where studies have not been conducted. The IDW method assumes that the prevalence of the mutant genotype near observed data points are the most similar with decreasing similarity as distance from the observed data point increases. The amount of influence a given data point has at a certain distance is also influenced by a power value (p) that is generated by the ArcGIS software. The software determines the optimal (p) value for the map by minimizing the root mean square prediction error (RMSPE), which is a summary statistic that describes the error of the prediction surface. A (p) value of "0" means a given data point has equal influence on all parts of the prediction surface regardless of the distance from the point. Whereas, a large (p) value means the influence of a data point drops off very quickly as the distance from the point increases. The optimal (p) value for each map is shown in Figures 1 and 2.

The prediction surface of each map is shaded using a gradient scheme consisting of quintiles. Areas of the prediction surface considered to have a high prevalence of mutant genotypes are denoted by darker shading, whereas areas considered to have a lower estimated mutant genotype prevalence are denoted by lighter shading. The IDW spatial representation does not account for the date of the study. Therefore, shaded areas of the maps can only serve as crude snapshots of mutant allele prevalence between 1993 and 2008 with decreasing reliability as distance from observed data points increases. The MARA [66] map describing malaria transmission in Africa was used to restrict the shading on the mutant genotype prevalence maps to areas where malaria transmission is known to be present.

**Figure 3:**
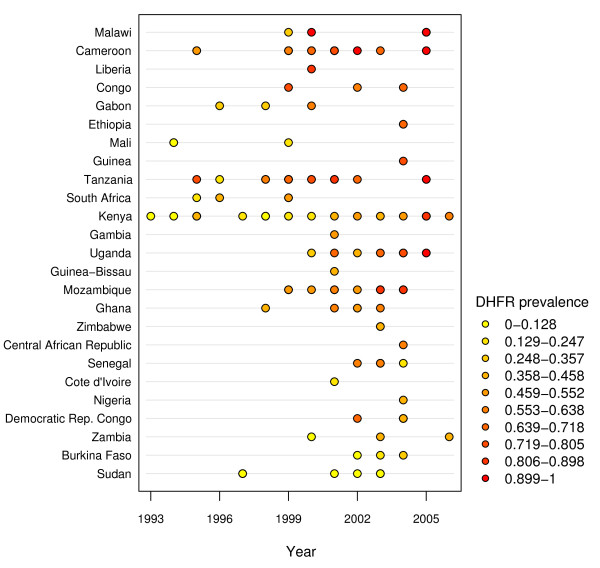
**Time plot of reported frequency - *dhfr *triple mutant genotype (N51I, C59R, S108N)**.

**Figure 4:**
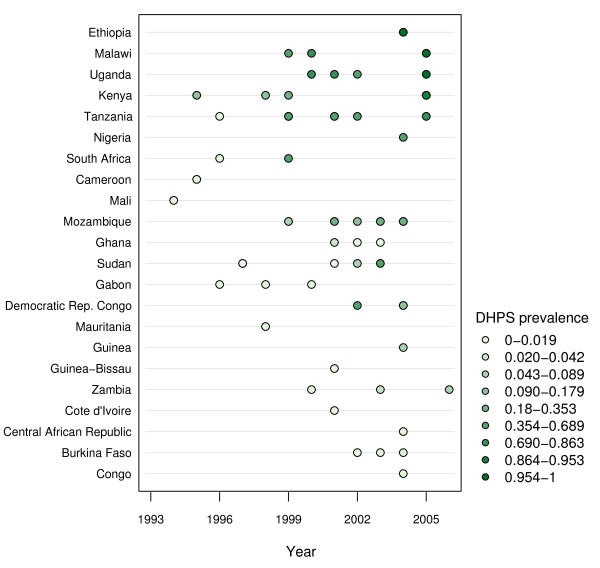
**Time plot of reported frequency - *dhps *double mutant genotype (A437G, K540E)**.

Time plots were created (see Figures 3 and 4) to supplement the maps by showing reported genotype frequencies from individual studies over time using a similar shading scheme. Each dot represents at least one study conducted for that year. The darker shaded dots on the time plot reflect higher mutant genotype prevalence, whereas the lower prevalence values are denoted by lighter dots. Multiple studies conducted in the same country and in the same year are still denoted by a single dot whose color represents a weighted average of the genotype frequency reported by each study. The time plots and maps were created separately because a geo-statistical map that accounts for several variables, similar to the world malaria map put forth by Hay *et al *[67], was not feasible given sample size limitations and an inability to directly compare studies due to the unknown impact of variables among molecular studies.

### Statistical analysis of reported mutant genotype frequencies and study variables

**Figure 5:**
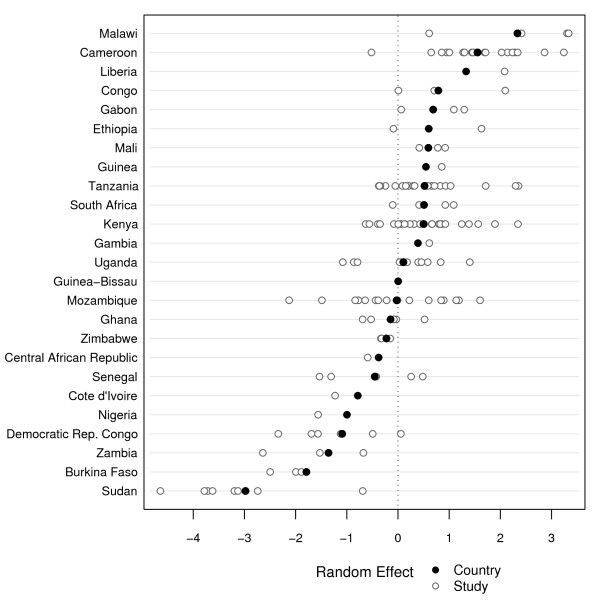
**Country and study level random effects - *dhfr *triple mutant genotype (N51I, C59R, S108N)**.

**Figure 6:**
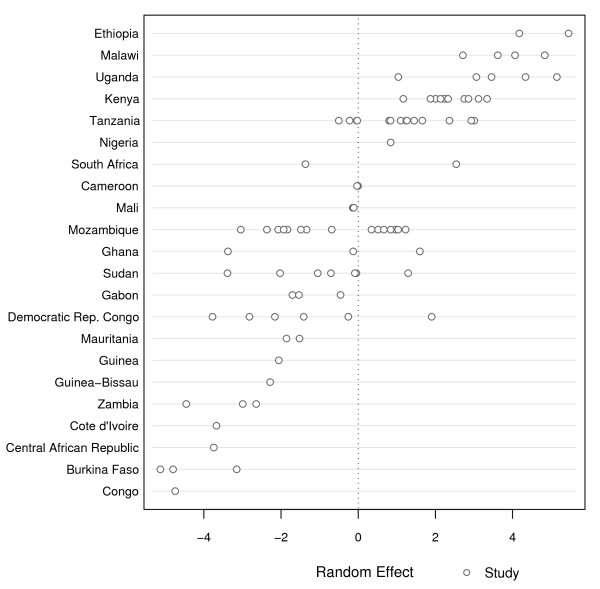
**Study level random effect - *dhps *double mutant genotype (A437G, K540E)**.

A multilevel mixed effects logistic regression model [68] is used to determine how factors including: location of study, year of study, type of study, age of sampled population, and mixed genotype categorization are associated with the reported frequency of the *dhfr *triple mutant and *dhps *double mutant genotypes in Africa. This belongs to the class of generalized linear mixed models which has been well established in both theoretical and applied academic literature [69,70]. Furthermore, such models have also been applied to meta-analysis of clinical or observational studies [71-73]. In the model for the *dhfr *triple mutant genotypes (Figure 5), the study and country of the study are included as nested intercept random effects in order to model the variability of the probability of mutation between studies within a country and also between countries. This induces a correlation between individuals within the same study and between studies within the same country. In the model for the *dhps *double mutant genotype (Figure 6), only the study random intercept effect is included due to inestimability of the country random effect. This still induces a correlation between individuals within the same study; however, in this model, studies within the same country are considered to be independent. The remaining factors: year of study, type of study, age of sampled population, and mixed genotype categorization are considered fixed effects in the regression model.

The year of study is centered at the year of the first study observed, 1993. Hence a study conducted in 1993 is given a year of 0, and a study conducted in 2005 is given a year of 12. This convention is used to facilitate interpretation of the intercepts as representing the probability of mutation at the start of the study time period (1993), and also helps to avoid convergence issues in fitting the model [74]. Year enters the model as a continuous variable; various categorizations of year were considered but deemed unnecessary. The type of study is a dichotomous variable that describes each study as either a clinical study or a survey. Studies that only sampled from a population of individuals symptomatic for uncomplicated malaria are considered clinical; whereas, studies that sampled from an asymptomatic population are considered surveys.

The fixed effect representing the age of sampled population are divided into five age categories: 1) adolescents and adults, 2) children and adolescents, 3) children under 6 years, 4) mixed ages, 5) N/A. For this study, individuals age 12 and under are considered children, between 12 and 18 years of age are considered adolescents, and age 18 or greater are considered adults. A study is assigned to age category 1, 2, or 3 if greater than 75% of the sampled population for that study fit the category description. If the age of the sampled population is described but did not meet the 75% threshold then it is assigned to the mixed ages category. The N/A category is used to describe studies that did not describe the age of the sampled population.

The fixed effect representing mixed genotype categorization is divided into four categories based on the methodology used for reporting mixed genotypes: 1) separate, 2) combined, 3) excluded, and 4) N/A. The separate category is assigned to studies that report mixed genotypes separate from pure mutant or pure wildtype genotypes. The combined category is used to describe studies that counted mixed genotypes as pure mutant genotypes. The excluded category is used to describe studies that completely removed mixed genotype data. The N/A category is used to describe studies that did not describe mixed genotypes in the study. The criteria used to categorize studies based on reporting of mixed genotype data is more thoroughly described in Table 3.

### Statistical model

Let *Y_ij _*be the number individuals with the mutant genotype in the *j^th ^*study of the *i^th ^*country. Conditional on the fixed and random effects, *Y*_*ij *_*~ Binomial*(*n*_*ij*_, *π*_*ij*_), where *n_ij _*is the number of individuals in the *j^th ^*study of the *i^th ^*country. On the logit scale, the probability of mutation *π_ij _*is modeled as a linear combination of the fixed and random effects. Equation (1) represents the logistic regression model for *dhfr *triple mutant genotypes, and equation (2) represents the logistic regression model for the *dhps *double mutant genotypes.

(1) $$\log it(\pi_{ij})=\alpha +v_{i}+u_{ij}+\beta x_{ij}$$

(2) $$\log it(\pi_{ij})=\alpha +u_{ij}+\beta x_{ij}$$

*i = *1, ..., number of countries; *j *= 1, ..., number of studies in the *i^th ^*country

Here, α is the fixed intercept, *v_i _*is the intercept random effect of the *i^th ^*country, *u_ij _*is the intercept random effect of the *j^th ^*study in the i*^th ^*country, and ***β ***is a vector representing the fixed study level effects. The study and country random intercepts allow the intercept to differ between studies. For model (1) the country random effects *v_i _*are assumed to be normally distributed *v_i _*~ *N*(0, σ_*v, dhfr*_^*2*^), while the study random effects *u_ij _*are assumed to be normally distributed *u_ij _~ N*(0, σ_*u, dhfr*_^*2*^). For the analysis of *dhfr *triple mutant genotypes, *σ*_*v, dhfr*_^*2 *^represents the heterogeneity between countries, and *σ*_*u, dhfr*_^*2 *^represents the heterogeneity between studies within a country. In model (2) the study random effects *u*_*ij *_are assumed to be normally distributed *u*_*ij *_~ *N(0, σ*_*u, dhps*_^*2*^), and σ_*u, dhps*_^*2 *^represents the heterogeneity between studies of the *dhps *double mutant genotypes. The variance components are estimated on the logit scale, and therefore do not have a direct interpretation. Statistical analysis was performed in R version 2.8.1 using the *lmer *function in the *lme4 *package, which uses Laplace approximation to the likelihood to obtain parameter estimates [75]. The significance of the fixed effects is evaluated through standard likelihood ratio tests, and the significance of the variance components for the random effects is evaluated through likelihood ratio tests where the asymptotic distribution of the likelihood ratio test statistic follows a mixture of chi-squared distributions [76]; *p*-values less than 0.05 are considered statistically significant.

## Results

### Literature search and databases

The literature search of the PubMed database yielded approximately 750 articles after search terms were applied. Of these 750 articles, most were excluded because they did not report the frequency of *dhfr *and/or *dhps *genotypes. Of the remaining articles, several were excluded on the basis of their location outside of Africa. Few studies were excluded on the basis of other criteria (ie. language of article not in English, travelers included in the sample population, and drug administration prior to molecular analysis). Data was successfully extracted from 100 articles that yielded genotype frequency information for 220 studies conducted in 31 African countries and published prior to 2009. Of these 220 studies, 207 studies reported the frequency of one or more mutations of interest within the *dhfr *or *dhps *gene. These mutations include *dhfr *A16V, C50R, N51I, C59R, S108N/T, I164L and *dhps *S436A/F, A437G, K540E, A581G, A613 S. Studies reporting the frequency of these individual mutations are summarized in the mutant allele frequency (MAF) database (Additional File 1). 161 studies reported the frequency of either the *dhfr *triple mutant genotype (N51I, C59R, S108N) or the *dhps *double mutant genotype (A437G and K540E), or both. These data are summarized in the multiple mutation (MM) database (Additional File 2).

Both the MAF and MGF databases are organized in alphabetical order by country. Each entry within the database reports mutant genotype frequency and descriptive information including: the location of the study, age of sampled population, categorization of mixed genotype data, and year the study was conducted. Study location is reported as specifically as possible and is typically identified by a recognizable city or village; however, in some cases, the location could only be described on the scale of a region or district. Most studies provided the age of the sampled population with a description of the age range. However, some also reported the mean or median age of the sampled population and these data are reported where available.

Mixed genotype categorization was based upon the convention used in the study to describe how mixed genotypes were considered when calculating genotype frequencies; however, in many cases mixed genotypes can be interpreted in multiple ways. For this review, the default convention was to split the mixed genotype data in the MAF database (Additional File 1) if the data permitted this interpretation. However, the default convention was to report mixed genotype data separately in the MGF database (Additional File 2) if the data permitted this interpretation.

The MAF and MGF databases also report the date the study was conducted. Studies were typically conducted within the span of a single year; however, a number of studies spanned a number of years. If a date range was provided, the last year of the study was used for statistical analysis. The sample size of the study is given in the MGF database; however, the sample sizes are not given in the MAF database because the number of isolates examined for each codon often varied.

### Maps and time plots of *dhfr *triple mutant and *dhps *double mutant genotypes

The maps shown in Figures 1 and 2 highlight two major qualitative features: 1) the distribution of studies across the continent and 2) the location of resistance hotspots.

The map describing the prevalence of the *dhfr *triple mutant genotype across Africa (Figure 1) shows the location of 159 studies. An examination of Figure 1 indicates a strong reporting bias in favor of certain parts of the continent. In fact, 43.9% of studies reporting the prevalence of *dhfr *triple mutant genotype were conducted in Kenya, Tanzania, and Cameroon alone. The red shading on the map indicates very high prevalence (> 70% of sampled isolates) of the *dhfr *triple mutant genotype in parts of Cameroon, Uganda, Kenya, Tanzania, and Malawi.

The map describing the prevalence of the *dhps *double mutant genotype across Africa (Figure 2) shows the location of 94 studies. The map also indicates that a large number of studies were conducted around Kenya and Tanzania; however, there are also several studies reporting these data in southern Mozambique. A large portion, 41.5% of total studies examining prevalence of the *dhps *double mutant genotype, was conducted in these three countries. Dark green shading in Figure 2 indicates high prevalence of the *dhps *double mutant in parts of Uganda, Kenya, Tanzania, and Malawi.

A comparison of the *dhfr *triple mutant genotype map and *dhps *double mutant genotype map indicates that both had a high prevalence in overlapping areas of East Africa; however, this is not the case in West Africa where the *dhps *double mutant genotype had a much lower prevalence. A comparison of the data shows that 56.1% of the isolates sampled in East Africa carry the *dhfr *triple mutant genotype and 44.3% carry the *dhps *double mutant genotype. However, in West Africa, 51.5% of sampled isolates carry the *dhfr *triple mutant genotype and only 3.0% carry the *dhps *double mutant genotype (see Table 4 for complete comparative analysis).

**Table 4:** Snapshot of data summarized by maps and time plots

	*dhfr *triple mutant genotype (N51I, C59R, S108N)	*dhps *double mutant genotype (A437G, K540E)
Number of studies	159	94
Mean year of study conducted	2000.79	2001.08
Std Dev year of study conducted	3.35	3.40
Percentage of studies in top three countries**^**	43.4%	41.5%
Percentage of studies conducted in East Africa†	67.3%	74.5%
Percentage of studies conducted in West Africa†	32.7%	25.5%
Number of total isolates examined	14316	7959
Average number of isolates sampled per study	91.2	84.7
Percentage of total isolates carrying genotype**	54.6%	33.7%
Percentage of isolates carrying genotype in East Africa†**	56.1%	44.3%
Percentage of isolates carrying genotype in West Africa†**	51.5%	3.0%

**^ **This value serves as an indicator for the clustering of studies: Top three countries reporting *dhfr *triple mutant genotype are: Kenya, Tanzania, and Cameroon; Top three countries reporting *dhps *double mutant genotype are: Kenya, Tanzania, and Mozambique

** Weighted average of genotype prevalence, raw data taken from (Additional File 2)

† East and West Africa were defined by the longitudinal line at 22.5°E

The time plots (Figures 3 and 4) show the reported frequency of both the *dhfr *triple mutant genotype and *dhps *double mutant genotype for studies reported since 1993, respectively. Both plots reveal an increase in the number of studies being conducted since 1993 that report the frequency of these mutant genotypes in Africa. Most studies were conducted around 2001, with a majority between 1998 and 2004. The shading of the points on the time plots suggests greater prevalence of both the *dhfr *triple mutant genotype and *dhps *double mutant genotype being reported in later studies in parts of Uganda, Kenya, and Tanzania. The reported frequency of both the *dhfr *triple and *dhps *double mutant genotypes in individual studies appears to be constant or even decreasing over time in a few countries. These perceived "trends" have little support since historical data is limited in these countries and the variation in reported frequency of mutant genotypes, even among studies within a single country, is high. Therefore, the time plots serve primarily as a summary of the limited data available and are not to be interpreted as an authoritative estimate of countrywide trends.

### Statistical analysis of mutant genotype prevalence and study variables

Multilevel mixed-effects regression was used to assess the association of several variables on the reported frequency of both the *dhfr *triple mutant genotype and the *dhps *double mutant genotype independently. For the *dhfr *triple mutant genotype, the results from 14,109 individuals in 155 studies from 25 countries were analyzed; the number of studies per country ranges from *j *= 1 to 26, and the size of the study ranges from *n*_*ij *_= 3 to 453. The regression model for the *dhfr *triple mutant genotype yielded study level and country level intercept random effects (see Figure 5). The x-axis in Figure 5 indicates the random effect value and is centered at zero, which is the theoretical mean of the country random intercepts. The country level random effect, *v_i _*(denoted by a dark filled circle), displays the variability of the probability of mutation between countries. Having a random intercept near zero implies that the study's country level random intercept in the logistic regression model is close to the overall fixed continent intercept, α. Countries with smaller random country effects are estimated to have a lower probability of mutation at the start of the study time period compared to countries with larger random effects. The study level random effects are nested within the country effects, and therefore are centered at the country level random effects. The study level random effects (denoted by a hollow circle) are displayed as *v_i _*+ *u_ij _*and show the variability of the probability of mutation between studies within the same country. The y-axis of Figure 5 is ordered by the country level random effect, from largest on top to smallest on the bottom. The regression confirms significant variation for both the study level (P <0.01) and country level (P <0.01) random effect, with variation between countries (1.56) larger than variation between studies within a country (0.89). This suggests that there is significant variability in mutant genotype frequency reported among different countries and among studies within a specific country.

For the fixed effects, the year of the study is significantly associated (P < 0.01) with the frequency of the *dhfr *triple mutant genotype and has a positive parameter estimate. Therefore, the model suggests that, conditional on the random effects, the reported frequency of this genotype in Africa is increasing over time. Of the three remaining variables that were examined, only mixed mutant categorization had a significant (P = 0.01) association with the reported frequency of the *dhfr *triple mutant genotype. The combined, excluded, and N/A categories all had a positive parameter estimates relative to the separate category; however, only the combined and excluded categories significantly differed from the separate category. This suggests that studies reporting mixed mutant genotypes as pure mutants or excluded them completely tend to have significantly higher reported frequency of the *dhfr *triple mutant genotype than studies that did not describe mixed mutant genotypes. The age of the sampled population and the type of study (clinical or survey) were not significantly associated with reported mutant genotype frequency. A summary of model results for the *dhfr *triple mutant genotype can be found in Table 5.

**Table 5:** Model results for *dhfr *triple mutant genotype

Variable	Estimate	Std. Error	P-value
Year	0.33	0.04	< 0.01
			
Type			
Survey	Referent	-	-
Clinical	-0.13	0.03	0.66
			
Age Category			0.66
Adolescents and adults	Referent	-	-
Children and adolescents	0.05	0.43	0.91
Children under 6	0.05	0.45	0.91
Mixed ages	-0.37	0.50	0.46
N/A	-0.43	0.53	0.41
			
Mixed Mutants			0.01
Separate	Referent	-	-
Combined	0.80	0.38	0.03
Excluded	1.11	0.40	0.01
N/A	0.21	0.32	0.50
			
Random Effects	Variance	Std Dev	P-value
Country	1.56	1.25	< 0.01
Study within Country	0.89	0.94	< 0.01

The model for the *dhps *double mutant genotype (see Figure 6) consisted of much fewer observations than the model for the *dhfr *triple mutant genotype. For the *dhps *double mutant genotype, the results from 7,761 individuals in 91 studies from 22 countries were assessed; the number of studies per country ranges from *j *= 1 to 15, and the size of the study ranges from *n*_*ij *_= 6 to 397. Two studies conducted prior to 1990 (one in Kenya and one in Zambia) were excluded from the statistical analysis because they were conducted earlier and could be too influential. A data shortage for the *dhps *double mutant genotype analysis led to convergence issues that did not allow for estimation of a country level random effect. However, the study level effect was still included in the model as a random intercept effect in order to allow for variation in the probability of mutation at the start of the study time period. As in Figure 5, the x-axis in Figure 6 indicates the random effect value and is centered at the theoretical value of zero. Figure 6 differs from Figure 5 in that a country level random effect could not be estimated in Figure 6; therefore, the y-axis of Figure 6 is ordered by the average study level random effect for each country, from largest on top to smallest on the bottom. Variation between studies for the analysis of the *dhps *double mutant genotype is highly significant (P < 0.01) and is estimated to be 8.83. This is much larger than the between or within country variation observed in the analysis of the *dhfr *triple mutant genotype because the variance for the *dhps *double mutant genotype could not be partitioned. This suggests that there is significant variability in the reported frequency of the *dhps *double mutant genotype among different studies at the start of the study time period.

The year of the study is significantly associated (P <0.01) with the reported frequency of the *dhps *double mutant genotype and has a positive parameter estimate. Therefore, the model suggests that, on average, mutant prevalence is increasing over time in Africa. The three remaining variables that were examined; mixed mutant categorization, age of sampled population, and type of study, were not significantly associated with the reported frequency of the *dhps *double mutant genotype. A summary of model results for the *dhps *double mutant genotype can be found in Table 6.

**Table 6:** Model results for *dhps *double mutant genotype

Variable	Estimate	Std. Error	P-value
Year	0.57	0.14	< 0.01
			
Type			
Survey	Referent	-	-
Clinical	-0.78	1.11	0.49
			
Age Category			0.22
Adolescents and adults	Referent	-	-
Children and adolescents	2.86	2.29	0.21
Children under 6	4.11	2.40	0.09
Mixed ages	3.90	2.34	0.10
N/A	4.21	2.53	0.10
			
Mixed Mutants			0.90
Separate	Referent	-	-
Combined	069	1.15	0.55
Excluded	0.74	1.13	0.51
N/A	0.42	1.06	0.69
			
Random Effects	Variance	Std Dev	P-value
Study	8.83	2.97	< 0.01

## Discussion

Broad inclusion criteria allowed most studies that report the frequency of *dhfr *and/or *dhps *genotypes in Africa to be included in this meta-analysis. The studies were conducted as early as 1993 and as late as 2006; however, the distribution is skewed (mean year: ~2001 for studies reporting either multiple mutant genotype) towards recent years reflecting an increase in the number of studies reporting the frequency of the *dhfr *triple mutant and/or *dhps *double mutant genotypes. This observation is in line with the increase in the number of anti-malarial clinical efficacy trials reported in the literature in recent years [77] and likely reflects the growing prioritization and feasibility of surveillance as a component of malaria control strategies. 161 studies assembled in this meta-analysis collectively examined blood isolates derived from over 14,000 individuals for the *dhfr *triple mutant genotype and nearly 8,000 individuals for the *dhps *double mutant genotype. The clear emphasis on reporting the *dhfr *triple mutant genotype is likely due to its emergence some 10-20 years earlier than the *dhps *double mutant genotype in Africa [78] and its ubiquitous presence across the continent. The *dhps *double mutant genotype (437G/540E), on the other hand, emerged much later in the early to mid-1990's [79] and is only prevalent in East Africa with a small number of cases reported in West Africa. The 436A/437G double mutant appears to be emerging in West Africa with apparent absence of 437G/540E double mutant genotype in some countries. The prevalence of individual 436A and 437G alleles have been summarized (see Additional File 1). However, the 436A/437G double mutant genotype was not independently summarized due to limited reporting of data on this genotype.

The maps describing the prevalence of the *dhfr *triple mutant genotype (Figure 1) and *dhps *double mutant genotype (Figure 2) illustrate a high concentration of studies in certain parts of the continent. This observation clearly demands an assessment of potential sources of reporting bias. A wealth of data in particular areas is due in part to the availability of research capacity in these areas to facilitate such studies. However, it is also due to molecular surveillance studies conducted more recently in some countries that provide a vast amount of data compared to traditional molecular studies that complement clinical efficacy trials. In fact, a majority of the multiple mutant genotype data available from Mozambique, Uganda, and Cameroon comes from just a few surveillance studies [80-83] that examined a large number of isolates from many sites within each country. Surveillance studies in Kenya [84] and Tanzania [63,85] also contributed a significant amount of molecular data relative to traditional molecular studies. Potential reporting and publication bias must also be considered when examining the snapshots of resistance hotspots in Figures 1 and 2. Areas where studies are concentrated appear to coincide with areas where there has been a higher reported frequency of the multiple mutant genotype. This may be due in part to bias in which molecular studies that show a high prevalence of mutant genotypes are more likely to be published than those that show little or no prevalence. Another potential explanation is that traditional molecular studies often complement clinical efficacy trials and are more likely to be conducted in areas where therapeutic failure rates are high and there is greater interest in the role resistance mechanisms may be playing.

This meta-analysis shows that there are some important differences in the reported frequency of the *dhfr *triple mutant genotype and *dhps *double mutant genotype. Specifically, the *dhfr *triple mutant genotype appears to be found in high prevalence in areas throughout the continent whereas the *dhps *double mutant genotype only appears to be prevalent in parts of East Africa. This observation corroborates findings published by Pearce *et al*, which also illustrated high prevalence of the *dhps *mutant genotype consisting of A437G, K540E in East Africa and low prevalence in West Africa. A number of factors such as the early emergence of the *dhfr *triple mutant genotype in Africa [78], pervasive drug pressure from the distribution of pyrimethamine salts for malaria prophylaxis in various parts of the world [86], and human migration patterns [87], may explain the greater distribution of this genotype throughout the continent. Another important difference between the *dhfr *triple mutant genotype and *dhps *double mutant genotype is the impact of varying reporting conventions for mixed genotype data. This study establishes that reporting conventions for mixed genotypes has a significant impact on the reported frequency of the *dhfr *triple mutant genotype. However, this was not observed for the reported frequency of the *dhps *double mutant genotype. This may be explained by differences in diversity for the *dhfr *and *dhps *genotypes found in Africa. Parasite populations that have greater diversity for a particular gene (ie. more than one genotype in high prevalence circulating in the population) are more likely to yield mixed genotypes than parasite populations that have a low diversity for a particular gene. Thus, differences in the way mixed genotypes are reported have a more pronounced effect on the prevalence rates reported for a gene that has greater diversity in a given population than one that does not. It has been shown that the *dhps *(A437G, K540E) mutant genotype is either ubiquitously present or absent in most of the populations where it has been examined [79]; therefore, polyclonal infections containing the *dhps *double mutant genotypes along with another *dhps *mutant genotype are less likely to occur. However, the *dhfr *triple mutant genotype has historically been found alongside a higher prevalence of sensitive, single, and double mutant *dhfr *genotypes circulating in the population [87,88]; therefore, mixed genotypes are more likely to occur, particularly in earlier studies before the triple mutant *dhfr *genotype became fixed in the population. This phenomenon may explain why reporting conventions for mixed genotype data was significantly associated with the reported frequency of the *dhfr *triple mutant genotype but not the *dhps *double mutant genotype.

The specific reasons responsible for the differential impact of reporting conventions for mixed genotype data on the reported frequency for either mutant genotype examined in this meta-analysis are difficult to identify and may also be an artifact of confounding variables that were not accounted for in the statistical regression used herein. Regardless, the fact remains that polyclonal infections commonly occur in many of the high transmission settings across Africa and the characterization of mixed genotypes is an issue that must be addressed. Novel methods for analyzing mixed infections [89], multivariate *in vitro *studies, and standardized interpretation of existing mixed genotype data [32] have all been proposed as methods to better characterize mixed genotypes and present promising avenues for the future. Although there are a number of differences in the distribution and prevalence of the *dhfr *triple mutant genotype and *dhps *double mutant genotype, there are also some similarities - The prevalence of both alleles is increasing on average in Africa. On average, the age and clinical status of the sampled population are not significantly associated with the reported frequency of either mutant genotype. This is in contrast to smaller regional studies that have shown association between age, treatment failure rate, and the prevalence of genetic markers for resistance [90,91]. The observations from this meta-analysis that suggest there is not a significant association between these factors should not be seen as a contradiction to more localized studies; rather, they testify to limitations that are inherent in meta-analyses, particularly in identifying regional trends. These limitations must be specifically addressed in order to contextualize the observations made in this study and appropriately articulate the conclusions that can be drawn from them.

One of the major limitations in this meta-analysis was the small number of variables that could be assessed in the statistical models. There are a number of clinical and epidemiological variables such as transmission, regional drug administration policies, and target populations (ie. pregnant women, co-infections, etc.) that may better explain the reported frequency of the *dhfr *triple mutant and *dhps *double mutant genotypes but could not be examined in this analysis. This was due to lack of power from limited datasets and inability to link these data to reported frequency data where this information was not specifically reported.

Another limiting factor that must be considered when making statements regarding trends across the continent is the confounding effect of space and time. The studies reporting the frequency of the *dhfr *and *dhps *multiple mutant genotypes included in this analysis were conducted over a period of 13 years. In most cases, the data from a particular country only spans a fraction of this 13 year period; therefore, there is a regional bias where data from a particular year is really a reflection of certain regions, not the continent as a whole. The sample size of studies included in the analysis may have also contributed to regional and temporal bias since the regressions were weighted. The mean study size was 90 isolates for studies reporting the *dhfr *triple mutant genotype and 85 isolates for studies reporting the *dhps *double mutant genotype. However, studies examined as few as 3 isolates and as many as 453 isolates (approximately 5% of the total number of studies had sample sizes of fewer than 15 isolates). Since studies that have small sample sizes are more likely to be skewed by rare events [92], and these smaller studies tend to occur in more remote areas, this means that remote areas may not be as accurately represented by reported frequency data. In light of these factors, it is more appropriate to consider the data conveyed by the maps and regressions as snapshots of cumulative mutant genotype data rather than a description of mutant genotype frequency at any specific point in space or time.

Finally, accessibility to studies and the interpretation of data from included studies that use different reporting conventions represented noteworthy sources of bias and error, respectively. The literature search in this study was limited to the Pubmed database and Cochrane Library. Molecular studies that were not available through these sources are not reported. Studies that were included still required a level of interpretation due to differences in methodological and reporting conventions; some of which were specifically examined in this meta-analysis. However, many of these aspects simply could not be addressed within the scope this study. Since there is no established format for the collection and dissemination of molecular data, there was difficulty in determining if molecular data for the same samples was published multiple times in different articles. Efforts were made to filter out articles that reported data from isolates that were already described in a different article; however, there was uncertainty for some articles and it is possible that molecular data for some isolates were reported twice and others were excluded unnecessarily.

## Conclusion

This database, despite its limitations, provides a centralized source for information describing the frequency of mutant *dhfr *and *dhps *genotypes in Africa that are known to confer anti-folate resistance. These data collectively show that the *dhfr *triple mutant genotype consisting of (N51I, C59R, S108N) is fixed in several parts of Africa and has been increasing, on average, over the time period from 1993 to 2006. The *dhps *double mutant genotype consisting of (A437G and K540E) also increased in prevalence, on average, during the same time period. However, this genotype appears to only be fixed in parts of East Africa while its prevalence in West Africa remains low. Continued drug pressure with SP may contribute to further selection of quadruple *dhfr *mutant genotypes and triple *dhps *mutant genotypes in Africa; similar to what has been observed in Asia and South America. The increasing prevalence and fixation of these mutant genotypes has historically been associated with a loss of anti-folate drug efficacy and may pose a threat to settings in Africa where anti-folates are still used to treat clinical malaria.

SP is currently used in sub-Saharan Africa for IPTp. In this context, the drug is used to clear *P. falciparum *infection in women with acquired immunity and usually low density asymptomatic parasitaemia. Thus, the treatment efficacy of SP for clinical malaria in young children cannot be directly extrapolated to its usefulness for IPTp [93]. However, a recent study by Harrington et al. [94] in Muheza, Tanzania shows that IPTp was associated with increased parasitemia and a higher degree of placental inflammation. The study also demonstrates selection of the resistance allele at *dhps *codon 581 in women receiving IPTp. In a subsequent study involving young children from Tanzania, emergence of triple mutant *dhps *allele with mutations in codons 437, 540 and 581 has been confirmed and this coincided with the high rate of therapeutic failure [95]. In the same report, preliminary data reporting the spread of parasites with *dhps *mutation at codon 581 to other parts of Africa has been presented. These findings suggest that the spread of the highly resistant triple mutant *dhps *allele along with triple/quadruple mutant *dhfr *alleles in different parts of Africa could further compromise the effectiveness of SP for the treatment of falciparum malaria and may have implications for IPTp. Therefore, systematic molecular surveillance studies coupled with assessment of drug effectiveness, including in pregnant women receiving IPTp, will be critical in the future to correlate molecular surveillance data with SP efficacy.

A continuation of efforts to conduct systematic molecular surveillance will further provide valuable information needed to construct future iterations of resistant genotype maps and databases; however, standardized methodology and reporting conventions are critical to improve the resolution and statistical power of centralized information systems. Challenges confronted during this meta-analysis suggest that patient specific data, standardized characterization of mixed genotypes, more specific date and locality descriptions, and better design and distribution of molecular studies are all critical objectives that must be pursued in order to fulfill the vision of the centralized resistance network proposed by WWARN.

## Competing interests

The authors declare that they have no competing interests

## Authors' contributions

VU and JWB conceived the original format and objectives of the meta-analysis. KMH produced an initial version of the database and genotype frequency maps. LMS and SS developed search criteria, data extraction methods, and tabular design. SS extracted and compiled the frequency data examined in the meta-analysis. SKM performed all statistical analyses and worked with SS and VU to write the first draft of the manuscript. All authors have read and approved the final draft of the manuscript.

## Supplementary Material

Additional file 1 **Mutant Allele Frequency (MAF) Database**. The data provided show the mutant allele frequency reported in each study, organized by country in alphabetical order Click here for file

Additional file 2 **Mutant Genotype Frequency (MGF) Database**. The data provided show the mutant genotype frequency reported in each study, organized by country in alphabetical order Click here for file

Additional file 3 **Article Database**. The data provided reference each article from which frequency data are extracted, organized by study identification number Click here for file
